# Methodology of electrical impedance tomography-derived measures of regional lung ventilation

**DOI:** 10.1186/s13054-014-0635-5

**Published:** 2014-11-18

**Authors:** Inéz Frerichs, Tobias Becher, Norbert Weiler

**Affiliations:** Department of Anaesthesiology and Intensive Care Medicine, University Medical Centre Schleswig-Holstein, Campus Kiel, Arnold-Heller-Str. 3, D-24145 Kiel, Germany

In the previous issue of *Critical Care*, we read with interest the article by Blankman and colleagues [1], who studied the performance of various electrical impedance tomography (EIT)-derived measures in detecting the ‘best’ positive end-expiratory pressure. The aim of that study is relevant; however, the article contains some methodological inaccuracies that need to be clarified.

One of the EIT measures used to characterize ventilation distribution is the center of ventilation (COV), first introduced in [2]. The authors refer appropriately to an article that contains a methodological figure illustrating how COV is derived from EIT ventilation images [3]. However, the authors incorrectly state that COV is the ratio between the EIT-derived ventilations in the dorsal and whole-image regions, provide a wrong equation 6, and attribute it to [3].

It is important for the understanding of EIT findings to appreciate that Figure one [1] does not show images of ‘impedance’ and the degree of ‘aeration’ (legend) but of tidal impedance differences representing regional tidal volumes. Regional ventilation delay (RVD) was analyzed not in [4], but in [5], where it was calculated in each pixel. A two-dimensional map was produced from these values, and standard deviation was calculated as an aggregate measure of ventilation homogeneity. Equation 3 regarding RVD calculation was used in [5], not in [6], where an additional multiplication by maximum impedance amplitude of the studied low-flow inflation was included. It is not clear which percentages of regional compliance are presented in Figure two B [1].

EIT is currently at an important stage of its development. Its clinical use might be fostered by implementation of accurate analysis tools.

## Abbreviations

COV: Center of ventilation
EIT: Electrical impedance tomography
RVD: Regional ventilation delay
